# Inhibition of lens-induced myopia in guinea pigs using a far-induced infrared ray material

**DOI:** 10.3389/fmed.2025.1545099

**Published:** 2025-03-31

**Authors:** Xu Weiwei, Liu Jingxi, Guoji Erti, Zhou Xinrun, Huang Yifei

**Affiliations:** ^1^Department of Ophthalmology, The Chinese PLA General Hospital, Beijing, China; ^2^National Research Institute for Family Planning, Beijing, China; ^3^School of Medicine, Nankai University, Tianjin, China

**Keywords:** myopia, far-infrared radiation (FIR), hypoxia, choroid, sclera, blood perfusion

## Abstract

**Purpose:**

Numerous studies have demonstrated a close relationship between choroidal thickness (ChT), sclera/choroidal hypoxia, and the onset and progression of myopia. Far-infrared (FIR) therapy is a traditional method used to enhance microcirculation. In this study, we estimated the effectiveness of FIR in myopia control and explored its underlying mechanisms. Furthermore, we compared the efficacy of FIR from two different sources in controlling myopia.

**Methods:**

Guinea pigs were divided into three groups, all of which underwent minus lens induction for 4 weeks. Two of the groups received simultaneous FIR intervention, either from a FIR radiator (FIRR) lamp or from an innovative FIR material (FIRM). Refraction, axial length (AL), ChT, and levels of hypoxia-labeled pimonidazole in the choroid and sclera were measured.

**Results:**

Both FIRR and FIRM inhibited increases in refraction and AL and attenuate the decrease in ChT. They also mitigated choroidal and scleral hypoxia. Compared to FIRR, FIRM demonstrated a greater effect on myopia control and hypoxia attenuation. However, the difference in AL reduction between the two FIR sources was not statistically significant.

**Conclusion:**

FIR effectively controls myopia, and the innovative FIR material may represent a breakthrough in myopia management in the near future.

## 1 Introduction

In recent years, the prevalence of myopia has rapidly increased. The global prevalence of myopia was approximately 30% in 2010 and is expected to increase to approximately 50% by 2050. In some regions of Southeast Asia, the prevalence of myopia among teenagers and young adults ranges from 86 to 97% (1). This is significantly higher than in North America, Europe, and the Middle East, where it is approximately 20–50% (2). Myopia is becoming an increasing global public health concern. In addition to the substantial socioeconomic costs, complications associated with high myopia—such as retinal detachment, myopic macular degeneration, and choroidal neovascularization—greatly compromise the quality of life (3, 4). Scleral extracellular matrix (ECM) remodeling plays a critical role in the development of myopia. Zhou et al. (5) demonstrated that exposure to hypoxia induced ECM remodeling in human scleral fibroblasts.

Choroidal thickness (ChT) changes correlate consistently with choroidal blood perfusion, which is closely associated with the development of myopia. The choroid is a highly vascularized structure that provides oxygen and nutrients to the adjacent retina and sclera (6, 7). The thickness of the choroid can rapidly change in response to blood flow. Numerous studies and clinical trials have (8–12) demonstrated that changes in ChT are positively correlated with changes in choroidal blood pressure (ChBP). Both factors are bidirectionally associated with the development of myopia and its recovery (13–15).

Zhou (16, 17) confirmed that reduced choroidal blood perfusion led to scleral hypoxia, ultimately resulting in myopia. They also demonstrated that increased choroidal blood perfusion decreased scleral hypoxia, thereby inhibiting the development of myopia. These results suggest new therapeutic strategies to control myopia. In their experiment, apomorphine and prazosin were administered daily via peribulbar injections to enhance choroidal blood perfusion. However, applying these treatments to humans is impractical.

Far-infrared (FIR) is a non-ionizing electromagnetic radiation with wavelengths of 4–16 μm (18). It can accelerate blood circulation (19), eventually leading to improved metabolism and enhanced transfer of chemical messengers (20). Photobiomodulation using low-energy photon irradiation in the far-red region has been used to treat several ophthalmological diseases, such as age-related macular degeneration (21, 22), retinitis pigmentosa (23), and amblyopia (24), yielding satisfactory results. This research aimed to assess whether far-infrared rays could inhibit lens-induced myopia (LIM) and to explore the underlying mechanisms. Another innovation involved utilizing a new material that could emit intense far-infrared rays at room temperature as the source of FIR. In this study, the effects and safety of this material for inhibiting LIM were also assessed.

## 2 Materials and methods

### 2.1 Experiment design

To determine whether FIR could inhibit LIM, far-infrared material (FIRM) and far-infrared radiator (FIRR) were administered (as described below) during the 4 weeks of LIM. Key parameters, including refraction and axial length (AL), were measured in all eyes before the experiment and after 4 weeks of minus lens induction paired with far-infrared radiation.

To explore the underlying mechanism, we measured the levels of ChT and hypoxia-labeled pimonidazole adducts in the choroid and sclera after 4 weeks of treatment. Pimonidazole hydrochloride is a hypoxia-sensitive imidazole that can form immunodetectable adducts with thiols in proteins, peptides, and amino acids in hypoxic cells (25).

### 2.2 Animals

Animal treatment and care were conducted in accordance with the Association for Research in Vision and Ophthalmology’s Statement on the Use of Animals in Ophthalmic and Vision Research. The study protocol was approved by the ethics committee in all participating centers (NO. jmy-20210307). The animals were kept on a daily cycle of 12 h of light (8:00–19:00), followed by 12 h of darkness, and the room temperature was maintained at 25°C.

### 2.3 Establishment of LIM models and far-infrared ray treatment

Three-week-old pigmented guinea pigs (*Cavia porcellus*, English short-haired stock, tricolor strain, n = 42) were randomly assigned to three experimental groups: two treatment groups and one corresponding control group. The treatments were administered as described below.

The right eye of all guinea pigs was selected as the experimental eye, while the left eye served as the untreated control. Each animal’s right eye was covered with a special facemask equipped with a − 10 diopter (D) lens at the eye location for 4 weeks to induce monocular myopia (26). During the 4 weeks of minus lens treatment, the LIM eyes and their fellow eyes in the treatment groups were subjected to far-infrared ray treatment. In the corresponding control group (LIM group), animals received minus lens treatment without any other intervention.

**Figure 1:**
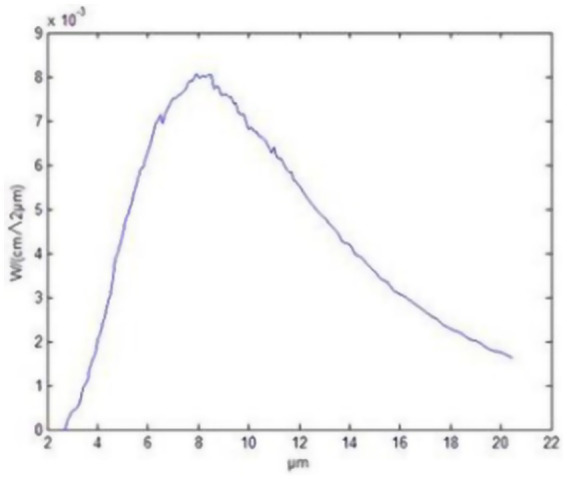
Far-infrared radiation pattern of Kaijingshi®.

In the present experiment, FIR material (FIRM) and FIR radiator (FIRR) were used to emit FIRs. The guinea pigs in the LIM + FIRM group (n = 14) were exposed to FIRs emitted by functional glass frames (Yidekai Technologies Co., Ltd., Beijing, China). These frames continuously emit intense FIR at room temperature (27). These functional glass frames are made of Kaijingshi®, an innovative material composed of microsized particles of tourmaline, rutile, and other minerals. The School of Mathematical and Physical Sciences at Beijing University of Science and Technology evaluated the full normal emissivity of Kaijingshi®, determining it to be 0.86, with a peak at 8–14 μm wavelengths (Figure 1). They also tested its safety, confirming that Kaijingshi® does not show toxicity. Tian et al. (27) conducted a clinical trial and reported on the effects and safety of these functional glass frames for the treatment of dry eyes. The functional glass frames were secured to the floor and ceiling of the rearing cage. The irradiance at the location of the animals was measured using a Schmidt–Boelter heat flux meter (Model No. GTW-10-32-485A, Medtherm Corp., Huntsville, AL, USA), resulting in a value of 0.71 kW/m^2^. The guinea pigs were raised in the “FIR functional glass frames” cage for 12 h (8:00–19:00) each day and moved to a normal cage during the remaining 12 h.

Animals in the LIM + FIRR group (*n* = 14) were exposed to far-infrared rays emitted by an FIR radiator (Model No. HP3621; Philips Consumer Lifestyle B.V., Germany). The emission wavelength of the FIR radiator was measured using a Fourier transform infrared spectrometer (Model No. Nicolet 6,700, Thermo Electron Corp., Madison, WI), ranging from 3 μm to 30 μm, with a peak at 10 μm. It was positioned 30 cm from the guinea pigs, and the radiation area measured 30 cm × 30 cm. The irradiance at the location of the animals was 0.75 kW/m^2^ when operated at an electric power of 200 W. The guinea pigs were exposed to this FIR radiator for 12 h each day (8:00–19:00). During the 12-h exposure, the radiation protocol consisted of 20 min of far infrared radiation followed by 2 min of normal light.

The range of wavelengths for FIR is 2 to 20 μm, with the emission peak of the ceramic material occurring at approximately 8 μm.

### 2.4 Biometric measurements

Before treatment, the baseline refraction and the AL of each eye were measured. After 4 weeks of treatment, the refraction, AL, and ChT were measured.

Refractive errors (spherical equivalent, SE) were measured using streak retinoscopy (Welch Allyn, Skaneateles Falls, NY, USA). All guinea pigs received cycloplegia with 5 mg/mL tropicamide (SINQI, China), which was administered once every 5 min for a total of three doses. Two researchers, Xu Weiwei and Zhou Xinrun, measured six times, and the average SE was used for analysis.

AL, measured using A-scan ultrasonography (Quantel Medical, France), represents the distance from the front of the corneal apex to the vitreoretinal interface. Before the measurement, the guinea pigs were lightly anesthetized with pentobarbital sodium (50 mg/kg). They were positioned to ensure the transducer probe was aligned centrally with the pupil along the optic axis. Six measurements were taken sequentially from each eye, and the average value was recorded.

**Figure 2:**
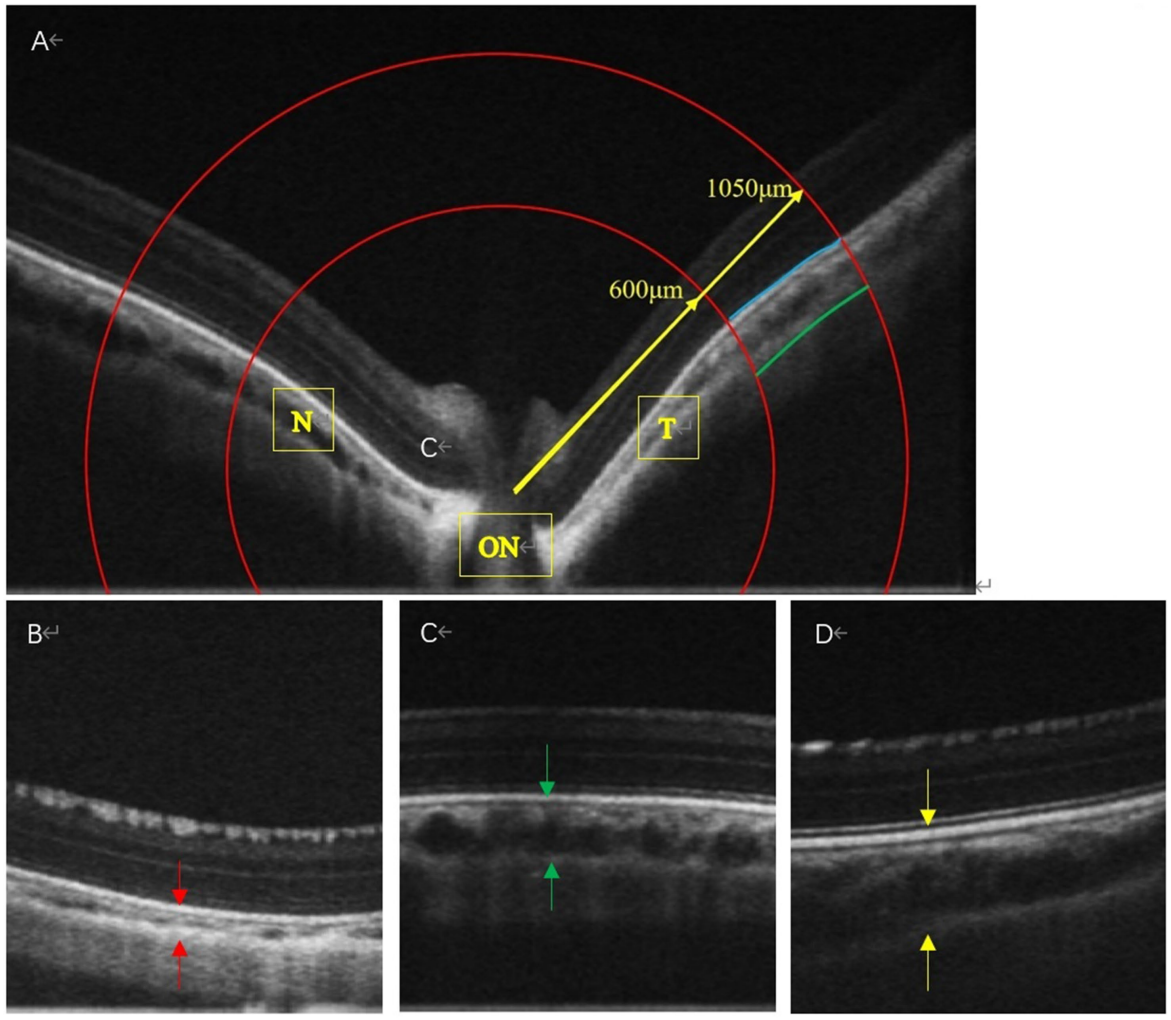
The OCT images of the LIM + FIRM, LIM + FIRR, and LIM groups The space between the two arrows is the choroid. N, nasal; T, temporal; ON, Optic Nerve; Each group has 14 guinea pigs.

ChT was measured using an Ultramicro Ophthalmological Imaging System (ISOCT, OPTOPROBE, Optoprobe Science Ltd., UK). After administering systemic anesthesia, carbomer eye gel (Bausch & Lomb) was applied before the ChT measurement. We selected scans along both the horizontal and vertical axes that passed through the center of the optic disc, with a pattern size of 30° × 15°. To ensure consistent measurements at a well-defined location in the fundus of the eye, we selected an area based on the optic disc. Using the optic disc as the center, we drew two concentric circles (Figure 2A, red lines) with radii of 600 and 1,050 μm (Figure 2A). The procedure adhered to the lateral magnification correction described by Howlett (28) and Jnawali (29). The choroid was defined as extending from the external surface of the retinal pigment epithelium to the internal surface of the sclera. Data were analyzed using OCT Image Analysis (version 2.0). Average values were calculated for the inner and outer circles at eight positions across the four quadrants. The final value for each eye was the average of three measurements. The choroid is a highly vascularized structure that provides oxygen and nutrients to the adjacent retina and sclera (6, 7). The thickness of the choroid can change rapidly in response to blood flow. Zhou et al. (8) demonstrated that changes in ChT were positively correlated with changes in ChBP. Therefore, in this study, we considered the dark area of the choroidal layer as an indication of blood flow in the vessels.

### 2.5 Immunofluorescent labeling of hypoxia signals

Hypoxic conditions of the choroid and sclera were assessed through the immunodetection of pimonidazole hydrochloride (25). Pimonidazole hydrochloride (Hypoxyprobe; Burlington, MA, USA) was injected into the inferior peribulbar space of both eyes. In total, 45 min later, the guinea pigs were anesthetized and euthanized. The eyeball was immediately isolated and sectioned along its equatorial plane. The posterior eyecup was fixed in 4% paraformaldehyde at room temperature for 30 min and dehydrated in 30% sucrose at 4°C for 24 h. The posterior eyecup was then embedded in mounting gel (Neg-50; Thermo Fisher Scientific, Waltham, MA, USA) and submerged in liquid nitrogen. It was sliced sagittally into sections (12 μm), followed by blocking with 10% normal donkey serum. The sections were incubated with the primary antibody, followed by the secondary antibody. The primary antibody used was rabbit anti-pimonidazole antibody (1:100; PAb2627AP, HPI), and the secondary antibody was fluorescein isothiocyanate-conjugated donkey anti-rabbit immunoglobulin G (1:400; Invitrogen, Waltham, MA, USA). Sections incubated without the primary antibody were used as negative controls. The sections were washed with PBS after each incubation step. A confocal microscope (LSM880 META; Carl Zeiss, Germany) was used to examine and acquire images. Matched software (Zeiss ZEN 2.3) was used to analyze the intensities of the immunofluorescence signal. The immunolabelings were assessed at the nasal and temporal sides (around the optic nerve) in each section. The mean intensity of the two scleral locations was used for analysis.

### 2.6 Statistical analyses

The data were expressed as means ± standard deviations. The normality of distribution and equality of variance were tested. Analysis of variance (ANOVA) was used to compare differences in refraction, AL, and ChT between the different groups. Independent t-tests were conducted to compare the intensities of the immunolabeling signals between pairs of different treatment groups. Multiple testing analyses were conducted using Bonferroni *post-hoc* correction. All statistical analyses were performed using IBM SPSS version 24.0 (IBM). Values of *p* < 0.05 were considered statistically significant.

## 3 Results

At the beginning of the experiment, there were no significant differences in refraction or AL between the different groups. After 4 weeks of minus lens-induced treatment, all right eyes that simultaneously received FIRM or FIRR treatments developed myopia, longer ALs, thinner ChTs, and greater choroidal and scleral hypoxic signals. Compared to the corresponding control groups (LIM group), the right eyes exhibited less myopia and had correspondingly shorter ALs, thicker ChTs, and lower choroidal and scleral hypoxic signals.

### 3.1 Far-infrared ray material and far-infrared ray radiator inhibited lens-induced myopia (LIM)

At the beginning of the experiment, there were no significant differences in refraction or AL between the right and left eyes of individual animals or among the LIM + FIRM, LIM + FIRR, or LIM groups. After 4 weeks of LIM, with or without simultaneous treatment, all right eyes developed myopia characterized by a lower spherical equivalent (SE), longer AL, and thinner ChT. Compared to the LIM group, eyes treated with FIRM and FIRR exhibited less myopia and shorter AL. As determined by repeated measures ANOVA, the main effects on refraction and AL were significant in both treatment groups.

**Figure 3:**
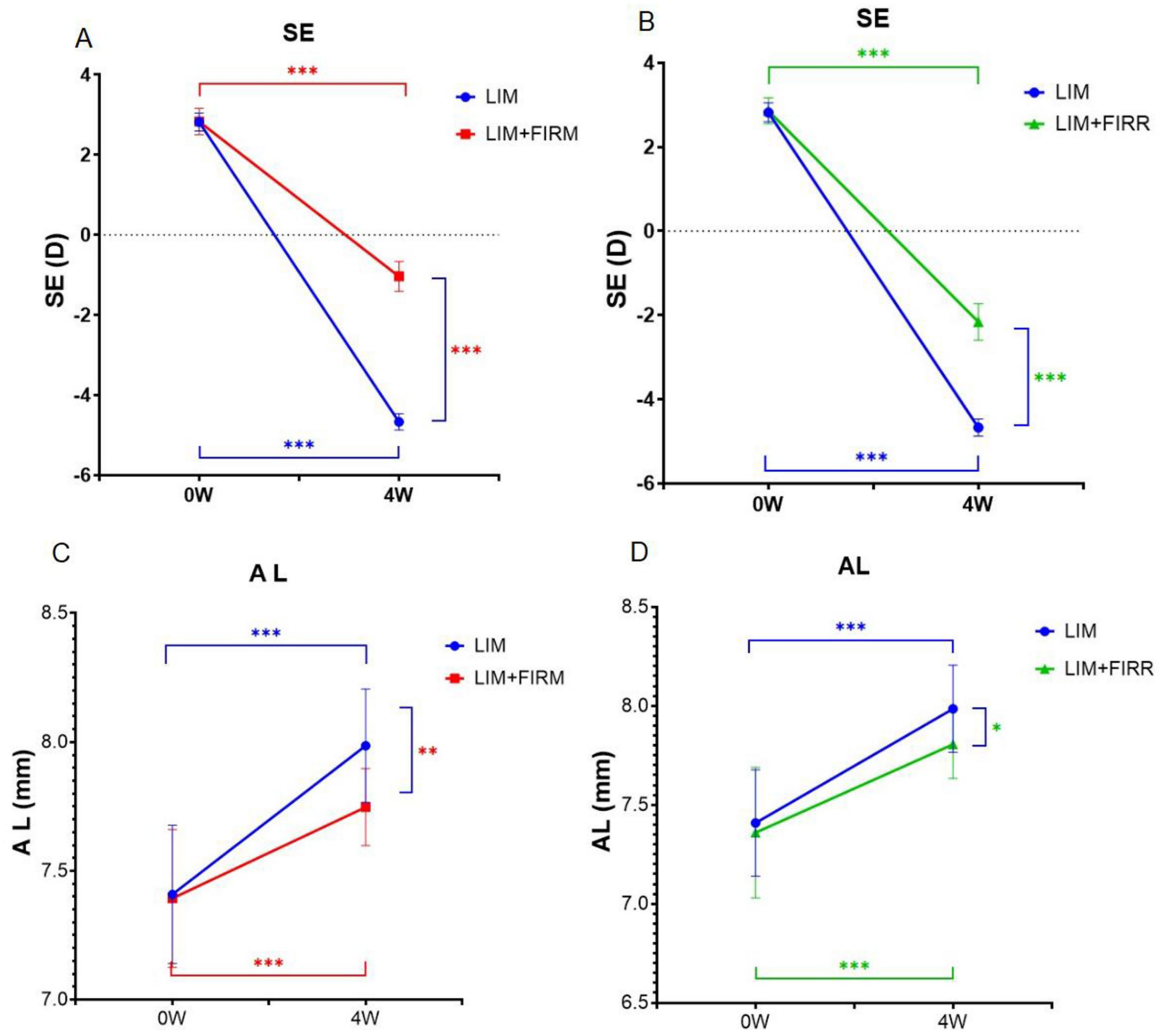
Refraction and AL for the LIM + FIRM, LIM + FIRR, and LIM groups. Comparison of the differences in refraction between LIM and LIM + FIRM group **(A)**. Comparison of the differences in refraction between LIM and LIM + FIRR group **(B)**. Comparison of the differences in AL between LIM and LIM + FIRM group **(C)**. Comparison of the differences in AL between LIM and LIM + FIRR group **(D)**. LIM + FIRR, and LIM groups at the beginning (0 W) and end (4 W) of the treatment period. W, weeks; 0 W, at baseline; 4 W, after 4 weeks of treatment; LIM, LIM eye without FIR treatment; LIM + FIRM, LIM eye with simultaneous far-infrared material radiation; LIM + FIRR, LIM eye with simultaneous far infrared radiator radiation; NS, nonsignificant; *p < 0.05, **p < 0.01, and ***p < 0.001. Every group has 14 guinea pigs.

After 4 weeks, the mean SE change for the right eye in the LIM + FIRM and LIM + FIRR groups was −3.87 ± 0.56 D and − 5.02 ± 0.65 D, respectively, both significantly lower than that of the LIM group, −7.49 ± 0.32 D (*p* < 0.001 for both comparisons) (Figures 3A,B). The mean AL change was consistent with the SE change. The mean AL change for the right eye in the LIM + FIRM and LIM + FIRR groups was 0.25 ± 0.23 μm and 0.45 ± 0.20 μm, respectively, which were significantly lower than that of the LIM group (0.58 ± 0.21 μm) (*p* = 0.021) (Figures 3C,D). After 4 weeks of treatment, the mean SE of the LIM + FIRM group (−1.034 ± 0.373 D) and the LIM + FIRR group (−2.161 ± 0.437 D) was significantly higher than that of the LIM group (−4.671 ± 0.205 D) (*p* < 0.001 for both comparisons) (Figures 3A,B). The mean AL of the LIM + FIRM group (7.75 ± 0.15 μm) and LIM + FIRR group (7.81 ± 0.17 μm) was significantly shorter than that of the LIM group (7.99 ± 0.22 μm) (*p* = 0.004 and *p* = 0.034, respectively) (Figures 3C,D).

### 3.2 FIR attenuated LIM-induced reductions in ChT

**Figure 4:**
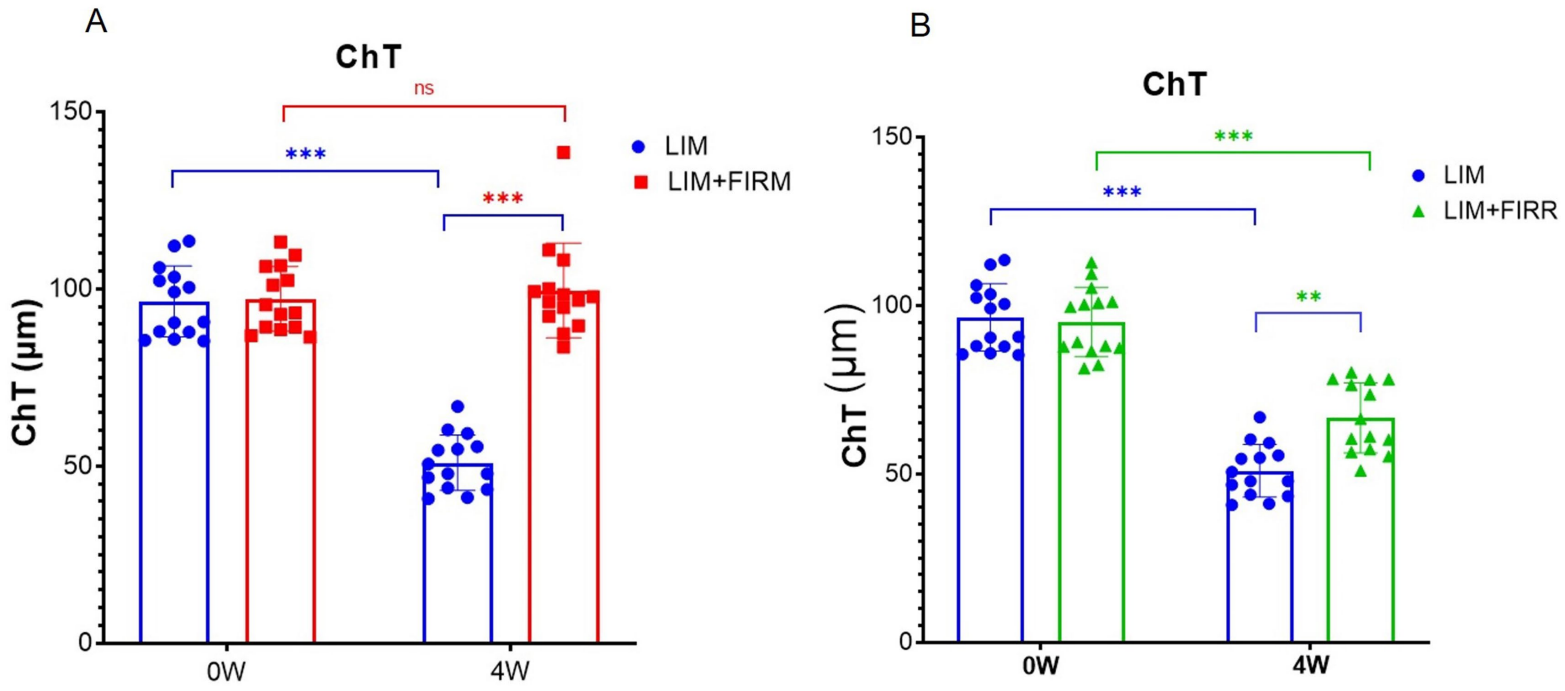
ChT of LIM + FIRM, LIM + FIRR, and LIM groups Comparison of ChT between the LIM + FIRM group and the LIM group **(A)**. Comparison of ChT between the LIM + FIRR group and the LIM group **(B)**. (0 W) and end (4 W) of the treatment period. W, weeks; 0 W, at baseline; 4 W, after 4 weeks of treatment; NS, nonsignificant; *p < 0.05, **p < 0.01, and ***p < 0.001. Each group has 14 guinea pigs.

During the horizontal scan (from the temporal quadrant to the nasal quadrant), we selected optical coherence tomography angiography (OCTA) scans that passed through the center of the optic disc (Figure 2A). The structural OCTA image showed the defined regions of interest: the inner choroid surface (blue line), the outer choroid surface (green line), and two concentric circles (red lines) at 600 μm (“in,” near the center of the optic disc) and 1,050 μm (“out,” away from the center of the optic disk). The region of interest in each quadrant was situated between the boundaries of the choroid layer and the two concentric circles. After 4 weeks of treatment, the mean ChT of the LIM + FIRM (99.55 ± 13.38 μm) and LIM + FIRR (66.60 ± 10.38 μm) groups was greater than that of the LIM group (50.93 ± 7.83 μm) (*p* < 0.001 and *p* = 0.001, respectively) (Figures 4A,B).

In the LIM group, the diameter of the choroidal vessels was very small, and the normal vessel structure could not be detected (Figure 2B). In the LIM + FIRM and LIM + FIRR groups, the choroidal morphology improved significantly. The diameter of the choroidal vessels was longer, and the vessel structure was clearly observable (Figures 2C,D). Compared to the LIM + FIRR group, this enhancement was more pronounced in the LIM + FIRM group (Figures 2C,D). The dark area of the choroidal layer indicated a longer diameter of the choroidal vessels, indicating rich blood perfusion.

### 3.3 Effects of refraction, AL, and ChT were more significant in the LIM + FIRM group

**Figure 5:**
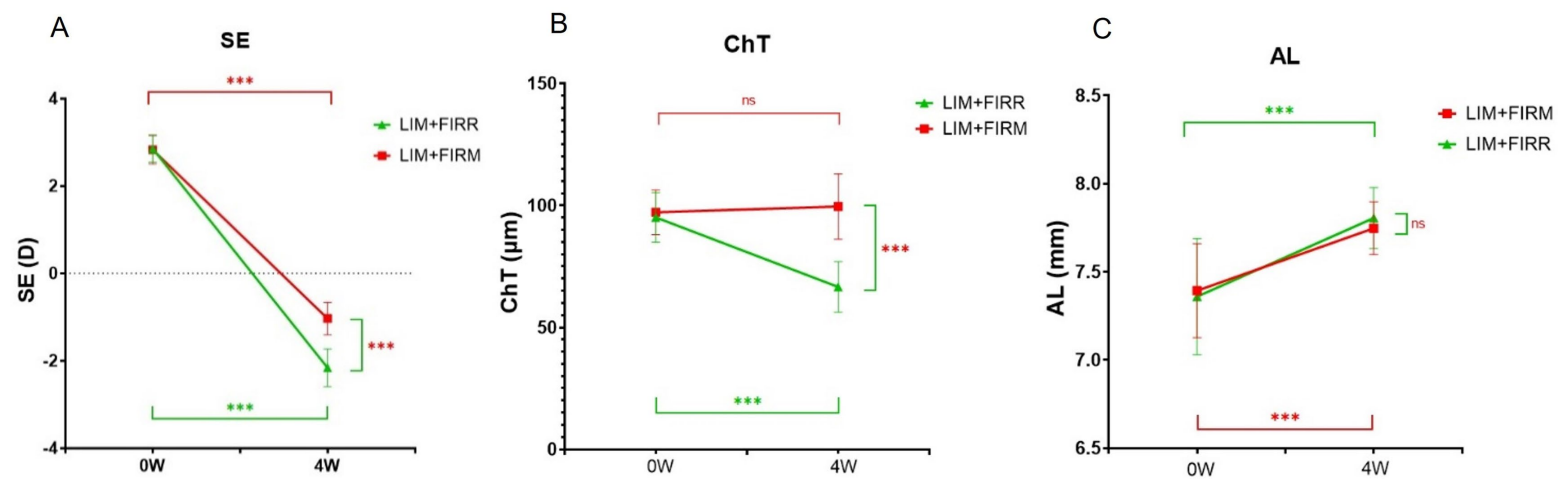
SE, ChT, and AL of the LIM + FIRM and LIM + FIRR groups Comparison of differences in SE **(A)**, ChT **(B)**, and AL **(C)** between the LIM + FIRM and LIM + FIRR groups at the beginning (0 W) and the end (4 W) of the treatment period. W: weeks; 0 W: at baseline; 4 W: after 4 weeks of treatment; NS: nonsignificant; *p < 0.05, **p < 0.01, and ***p < 0.001. Each group has 14 guinea pigs.

At the end of 4 weeks, the mean SE of the LIM + FIRM group (−1.03 ± 0.37 D) was higher than that of the LIM + FIRR group (−2.16 ± 0.44 D) (*p* < 0.001) (Figure 5A). Similarly, the mean ChT of the LIM + FIRM group (99.55 ± 13.38 μm) was greater than that of the LIM + FIRR group (66.60 ± 10.38 μm) (*p* < 0.001) (Figure 5B). The mean AL of the LIM + FIRM group (7.75 ± 0.15 μm) was slightly shorter than that of the LIM + FIRR group (7.81 ± 0.17 μm), but no statistical significance was found between the two groups (*p* = 0.677) (Figure 5C).

### 3.4 FIRM and FIRR attenuated scleral and choroidal hypoxia induced by LIM, with a more significant effect observed in the FIRM group

**Figure 6:**
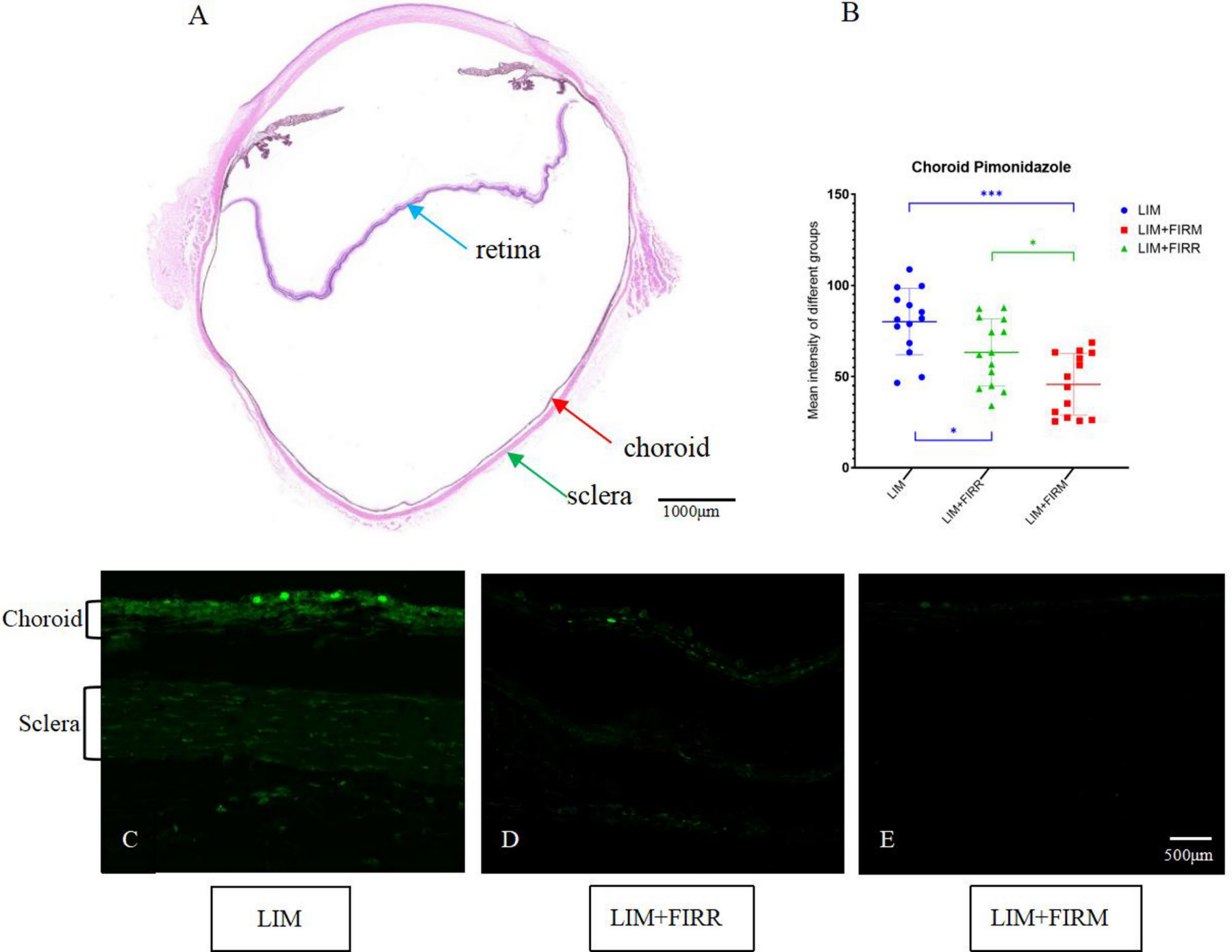
Hypoxia-dependent choroidal labeling using pimonidazole for the LIM + FIRM, LIM + FIRR, and LIM groups. **(A)** HE sections of the whole eyeball. The blue, red, and green arrows showed the retina, choroid, and sclera, respectively. The nasal and temporal locations around the optic disc were assayed. **(B)** After 4 weeks of treatment, the mean intensity of pimonidazole staining in the choroid and sclera of different groups. **(C–E)** Pimonidazole staining of the three groups. NS, non-siganificiant; *p < 0.05; **p < 0.01;***p < 0.001. Each group has 14 guinea pigs.

The hypoxic state of the choroid and sclera was indicated by pimonidazole labeling. Compared to the LIM + FIRM and LIM + FIRR groups, the intensity of pimonidazole labeling was significantly greater in the LIM eyes. This increase was inhibited by 4 weeks of far-infrared radiation (Figures 6C–E). The fluorescence intensity of the LIM + FIRM group (45.67 ± 16.79) and the LIM + FIRR group (63.26 ± 18.30) was significantly lower than that of the LIM group (80.09 ± 18.19) (*p* < 0.001, *p* = 0.043) (Figure 6B). Furthermore, the difference between the LIM + FIRM and LIM + FIRR groups was also significant, *p* = 0.033 (Figure 6B).

### 3.5 Complication

After 4 weeks of treatment, we used a slit lamp to observe the cornea and lens of the guinea pigs and did not find any cataracts, corneal ulcers, or other severe complications.

## 4 Discussion

In our study, we used FIR radiation to enhance choroidal blood perfusion. FIR is a conventional method for facilitating microcirculation. These mechanisms include promoting nitric oxide production (30), elevating the levels of calcium-regulated proteins, and reducing the production of cyclooxygenase-2 and prostaglandin E2 (31). FIR has been widely employed as a safe and non-invasive treatment for various diseases (32, 33). Wang et al. found that FIR radiation could promote neurite outgrowth in neuron-like PC12 cells (34). Fukui et al. (35) used far-infrared light to treat Alzheimer’s disease-transgenic mice and achieved satisfactory results. There are also some clinical studies that reported the effects of FIR radiation in treating myocardial ischemia, circulation issues, diabetes, kidney disease, and cancer (20, 36–38).

The traditional FIR therapy lamp raises the local temperature; therefore, it was placed 20–30 cm above the focus. After several minutes of irradiation, the lamp was moved away to prevent thermal burns. FIR therapy lamps are impractical for myopia control due to the potential risks of corneal damage and cataracts. Additionally, this therapy requires frequent placement and removal of the lamp, which significantly disrupts the patient’s normal daily life.

In this research, a functional far-infrared material was used to emit far-infrared rays for myopia control. It can emit intense FIR at room temperature (27). Tian’s research demonstrated its efficacy and safety for clinical use (27). Theoretically, Kaijingshi can be molded into any shape, allowing it to be crafted into a glass frame for myopia management. Compared to the FIR therapy lamp, the Kaijingshi® glass frame does not cause an increase in local temperature and can be worn continuously. Patients with myopia do not require additional time for treatment. As long as they wear the glass frame (with or without an ophthalmic lens), this far-infrared radiation material can continually influence the eyeball. It does not require additional therapeutic procedures and enables patients to receive treatment easily and consistently.

After 4 weeks of intervention, compared to the LIM group, the FIRM and FIRR groups showed higher SE degrees, shorter AL (Figure 3), and thicker ChT (Figure 4). These results indicate that both methods inhibit LIM and attenuate the decrease in ChT. The results of the choroid and scleral hypoxia test were consistent with the findings above. The maximum intensity of immunofluorescent labeling was detected in the LIM group, which was significantly stronger than that of the LIM + FIRM group and the LIM + FIRR group (Figure 6). This suggests that myopia leads to choroidal and scleral hypoxia, and this effect could be inhibited by FIR radiation. Compared to the LIM + FIRR group, the intensity of immunofluorescent labeling was weaker in the LIM + FIRM group (Figure 6). We can infer from these results that FIRM exhibits a superior effect in attenuating choroidal and scleral hypoxia compared to FIRR.

Between LIM + FIRM and LIM + FIRR groups, the mean SE and ChT of the FIRM group were significantly higher than those of the FIRR group at the end of the 4-week intervention. However, the mean AL showed no significant difference between the two groups (*p* > 0.05) (Figure 5). We hypothesize that the AL measurement method may need improvement to enhance measurement accuracy. In future studies, it is important to implement a longer intervention period to evaluate the effects of AL changes.

Several myopia control treatments are designed based on peripheral retinal defocusing mechanisms. Examples of these include orthokeratology (39, 40), multifocal contact lenses (41), bifocal lenses, and peripheral defocus lenses (39). However, the effects and safety of these options remain uncertain and are subject to debate (42–47). With the understanding of the mechanism of myopia, a series of studies have revealed a close relationship between myopia development and scleral/choroidal hypoxia (9, 16, 17, 48). The “myopia and hypoxia” theory has attracted extensive attention worldwide. In our study, we found that FIR inhibited LIM and attenuated scleral and choroidal hypoxia. We also determined that the innovative far-infrared material was more effective in controlling myopia compared to traditional FIR therapy lamps.

## Data Availability

The raw data supporting the conclusions of this article will be made available by the authors without undue reservation.
